# Genomics of the Genus *Rhodiola*: From Genome Assembly to High-Altitude Adaptation and Bioactive Compound Biosynthesis

**DOI:** 10.3390/genes17101163

**Published:** 2026-09-22

**Authors:** Mingcheng Wang

**Affiliations:** 1 Institute for Advanced Study, Chengdu University, No. 2025 Chengluo Road, Chengdu 610106, China; wangmingcheng@cdu.edu.cn; 2 Sichuan-Xizang Medicinal Resource Breeding and Standardization Team, Engineering Research Center of Sichuan-Xizang Traditional Medicinal Plant, Chengdu University, Chengdu 610106, China

**Keywords:** genus *Rhodiola*, genome evolution, stress adaptation, glycoside biosynthesis, multi-omics integration

## Abstract

**Background/Objectives**: The genus *Rhodiola* comprises alpine medicinal plants renowned for extreme high-altitude adaptability and valuable bioactive compounds. This narrative review synthesizes recent genomic advances to understand how genomic mechanisms contribute to both environmental adaptation and bioactive compound biosynthesis. **Methods**: This review is a narrative synthesis of genomic studies from the past decade, including chloroplast and nuclear assemblies, comparative genomics of transposable elements and gene families, and integrative multi-omics approaches for pathway elucidation. **Results**: Chloroplast genomes are structurally conserved but harbor sufficient variation for phylogenetic resolution. Nuclear genomes reveal dynamic patterns of expansion and contraction associated with transposable element bursts and lineage-specific gene family dynamics, although causal relationships remain to be established. Multi-omics integration has fully elucidated the salidroside pathway, identified candidate genes for rosavin biosynthesis, predicted transcriptional regulators, and suggested plant-microbiome contributions to specialized metabolism. **Conclusions**: These findings support *Rhodiola* as a promising model for studying coordinated high-altitude adaptation and bioactive compound biosynthesis. Future pan-genome construction, synthetic biology, and molecular breeding offer avenues for sustainable utilization and precision improvement of this medicinal genus.

## 1. Introduction

The genus *Rhodiola* L., belonging to the family Crassulaceae, encompasses a group of perennial herbaceous plants with considerable medicinal value [1]. With approximately 90 species distributed globally, the genus is widely found across the alpine and arctic regions of the Northern Hemisphere, and the Qinghai-Tibet Plateau (QTP) and its adjacent areas represent its modern center of distribution and diversification [2]. *Rhodiola* species exhibit remarkable habitat adaptability, colonizing a diverse array of high-altitude environments, including understory forests, shrub margins, alpine meadows, and alpine scree slopes, with a particular preference for open and rocky substrates [3]. The dramatic uplift of the QTP and the climatic oscillations of the Quaternary period have been identified as key drivers of the rapid radiation and diversification observed in this genus, positioning it as an exemplary taxon for investigating the adaptive evolution of plants in high-altitude environments [4,5,6].

In traditional medicine systems, *Rhodiola* species, particularly their roots and rhizomes, have a long history of therapeutic use [7]. The classic Tibetan medical text *Four Medical Tantras* describes *Rhodiola* as possessing properties that clear heat, counteract toxins, and replenish vital energy and blood. In Tibetan medicine, *Rhodiola* is recognized as one of the most frequently used herbs for treating pulmonary disorders [8]. Modern pharmacological research has demonstrated that the principal bioactive constituents of *Rhodiola* include salidroside, tyrosol, flavonoids, and other polyphenolic compounds [7,9]. These metabolites confer a broad spectrum of pharmacological activities, including notable anti-hypoxic, anti-fatigue, antioxidant, anti-aging, and adaptogenic effects [10,11,12]. In particular, salidroside mediates its anti-hypoxic effects through the modulation of multiple signaling pathways, such as the HIF-1α pathway, thereby alleviating hypoxic injury in various organs and tissues [13]. Consequently, *Rhodiola* holds considerable value in the prevention and treatment of high-altitude sickness and related disorders [1].

The genus *Rhodiola* uniquely integrates extreme environmental adaptability with exceptional medicinal value, raising a fundamental question: what genomic mechanisms have simultaneously enabled the adaptation of *Rhodiola* to extreme high-altitude environments and programmed the biosynthetic pathways responsible for its characteristic medicinal metabolites? Addressing this question holds significant implications for understanding the molecular basis of plant adaptive evolution as well as for guiding the sustainable utilization of medicinal plant resources [14,15]. In recent years, the rapid advancement of genomics, high-throughput sequencing technologies, and integrative multi-omics approaches has progressively redirected *Rhodiola* research from traditional morphological characterization and pharmacological evaluation toward a comprehensive elucidation of its genetic blueprints and molecular mechanisms [16,17]. Accumulating multi-layered data, ranging from plastid genomes to chromosome-level nuclear assemblies and from transcriptomes to metabolomes, now provide unprecedented opportunities to address the central questions outlined above.

This review provides a narrative synthesis of recent advances in genomic research in the genus *Rhodiola*. We first discuss chloroplast genome-based phylogenetic reconstruction and adaptive evolution. We then focus on breakthroughs in nuclear genome assembly and the “expansion-and-contraction” evolutionary pattern they have revealed. Finally, we explore how integrative multi-omics approaches have facilitated the discovery of key genes involved in bioactive compound biosynthesis and stress responses. Drawing upon available evidence, we propose an interpretative framework rather than a demonstrated mechanism: the coordinated evolution of high-altitude adaptation and bioactive metabolite accumulation in *Rhodiola* may be shaped by a combination of genomic conservation and localized genetic innovation. In this framework, genomic conservation is illustrated by the structural and sequence stability of chloroplast genomes, whereas localized genetic innovation is proposed to include repeat sequence bursts, gene family differentiation, and structural variations (SVs) within nuclear genomes. We acknowledge that causal links remain to be experimentally validated. Through this synthesis, we seek to present a unified picture that spans from genomic sequences to diverse ecological and pharmaceutical traits, while offering perspectives for future research directions.

## 2. A Three-Step Overview of Genomic Research in *Rhodiola*

The overall structure of this review is summarized in Figure 1, which outlines the three-step framework from chloroplast genomics to nuclear genomics and multi-omics integration.

### 2.1. Step One: Phylogeny and Adaptive Evolution Through Chloroplast Genomes

Over the past decade, chloroplast genome data have greatly advanced our understanding of phylogenetic relationships, adaptive evolution, and species identification in the genus *Rhodiola*. A series of studies with progressively expanded sampling and increasing analytical depth have systematically examined the structure, genomic variation, and phylogenetic utility of *Rhodiola* chloroplast genomes. In a pioneering study, Zhao et al. (2020) sequenced 12 representative species and seven outgroups, providing the first chloroplast genome-level dataset for the genus [18]. Subsequently, Zhao et al. (2021) sampled 23 species representing all subgenera and sections, employing chloroplast genome data to infer the genus’s spatiotemporal diversification [19]. Zhao et al. (2022) sequenced six additional species and screened chloroplast genomes for highly variable regions as candidate DNA markers [20]. Liu et al. (2024) assembled 23 chloroplast genomes from 18 species, uncovering novel compound repeat structures across the genus [6]. Huang et al. (2025) integrated nuclear and chloroplast data from 42 species and reclassified *Pseudosedum lievenii* into the genus *Rhodiola* [21]. Most recently, Zang et al. (2026) assembled 89 chloroplast genomes from 35 species, considerably expanding the taxonomic and population-level sampling coverage of the genus [22].

#### 2.1.1. Conservation and Diversity of Chloroplast Genomes

The chloroplast genomes of *Rhodiola* species possess the typical quadripartite circular structure characteristic of most angiosperms (Figure 2a), comprising a large single-copy (LSC) region, a small single-copy (SSC) region, and two inverted repeat (IR) regions [18]. Genome size is highly conserved across the genus, ranging from 150,286 bp in *Rhodiola smithii* to 152,136 bp in *R. rosea* [18,22]. The overall GC content varies narrowly between 37.7% and 37.8%, with the IR regions exhibiting markedly higher GC content than the LSC and SSC regions, a pattern consistent with the presence of GC-rich rRNA genes within the IRs [23]. Gene content and order are also largely conserved. A typical chloroplast genome comprises 134 genes, including 85 protein-coding genes, 37 tRNA genes, 8 rRNA genes, and four potential pseudogenes (*ycf1*, *rps19*, and two copies of *ycf15*); after accounting for duplicated copies in the IR regions, this leaves 114 unique genes. Minor variations in gene number have been reported in a few assemblies [6], which may be attributable to differences in annotation methods. Notably, no gene rearrangements or inversions have been detected within the genus, further underscoring the structural stability of *Rhodiola* chloroplast genomes.

Although the overall structure is conserved, variations are observed in the boundary regions between the IRs and the single-copy regions. For example, the IRB/SSC (JSB) and SSC/IRA (JSA) borders show slight shifts due to length polymorphisms at the 3′ ends of *ndhF* and *ycf1*. However, these shifts are typically minor and do not disrupt the general genomic architecture [6,18,22]. In addition, several highly variable regions have been reported, including the coding genes *ycf1*, *rps15*, *ndhF*, and *matK*, as well as the intergenic spacer *trnH*-*psbA* [20,22]. These regions exhibit elevated nucleotide diversity and are considered promising candidates for DNA barcoding, species delimitation, and phylogenetic studies within the genus. In a recent large-scale investigation, a rare 108-bp insertion in the ndhA-ndhH intergenic region was detected exclusively in *R. discolor*, constituting a novel structural variant [22]. Additionally, lineage-specific polymorphisms in *rps15*, *ndhI*, *rps3*, and *rpl20* have been linked with maternal lineage divergence and are suggested to correlate with sexual system differentiation, although their functional significance remains to be elucidated [22]. Collectively, these findings indicate that while *Rhodiola* chloroplast genomes are generally conserved, they nevertheless harbor sufficient variation to provide informative markers for evolutionary and taxonomic studies (Figure 2b).

#### 2.1.2. Rapid Radiation and Phylogenetic Reconstruction

Resolving phylogenetic relationships within rapidly radiating lineages has long posed a challenge due to short internal branches characterized by limited informative sites [24]. *Rhodiola* is widely recognized as a classic example of such rapid radiation on the QTP and adjacent mountain systems [4,5]. However, traditional markers, including the internal transcribed spacer (ITS) and a limited number of plastid fragments, have proved inadequate for resolving its backbone phylogeny, often resulting in polytomies and low support at key nodes [25]. In response to these challenges, chloroplast genome data have offered a powerful solution. By offering a substantially larger number of informative sites, chloroplast genomes have significantly improved phylogenetic resolution within the genus (Figure 2b).

The first chloroplast genome-scale phylogeny of *Rhodiola* established a robust framework for the genus [18]. All *Rhodiola* species formed a well-supported monophyletic clade sister to *Phedimus*, and the genus was consistently recovered as two major lineages: one composed predominantly of hermaphroditic species and the other exclusively of dioecious taxa [18]. This bipartite structure was subsequently corroborated through expanded taxon sampling [19]. Divergence time estimation placed the split between the two major clades at approximately 6.34 million years ago (late Miocene), a period coinciding with the rapid uplift of the Hengduan Mountains and the intensification of the Asian monsoon [19]. Ancestral area reconstruction further suggested that *Rhodiola* likely originated in the QTP and the Hengduan Mountains, with subsequent dispersal events to East Asia, Central Asia, Europe, and North America, a pattern consistent with an “out-of-QTP” migration model [4,19].

Beyond the conserved plastid-based phylogeny, recent phylogenomic and population-scale investigations have revealed more complex evolutionary scenarios. Cross-genomic comparisons integrating nuclear and chloroplast data from 42 species showed extensive cytonuclear discordance: the chloroplast tree partitioned *Rhodiola* into two primary lineages, whereas the nuclear tree recovered a successive branching pattern of six major clades [21]. This discordance was predominantly driven by recurrent hybridization and introgression, highlighting the necessity of nuclear data for capturing reticulate evolutionary histories, rather than relying on incomplete lineage sorting alone. Complementary evidence from 89 plastomes across 35 species revealed lineage-diagnostic polymorphisms in *rps15*, *ndhI*, *rps3*, and *rpl20* that consistently distinguished hermaphroditic from dioecious lineages, marking these loci as potential markers of maternal lineage divergence associated with sexual-system differentiation [22]. Collectively, these findings underscore that a full understanding of *Rhodiola* evolution demands integrating nuclear and plastid genomic data to capture both reticulate processes and lineage-specific variation.

### 2.2. Step Two: Insights from Nuclear Genomes

Nuclear genome assembly in *Rhodiola* has evolved from draft scaffolds to chromosome-level and near telomere-to-telomere (T2T) assemblies over the past decade, facilitating investigations into genome evolution, adaptive mechanisms, and specialized metabolite biosynthesis. Five key studies have documented milestones in this progression (Table 1). Fu et al. (2017) provided the first draft genome of *R. crenulata* at the scaffold level [26]. Zhang et al. (2024) reported the first chromosome-level genome assemblies for *Rhodiola*, representing a hermaphroditic species (*R. chrysanthemifolia*, 2n = 14) and a dioecious species (*R. kirilowii*, 2n = 22), and performed comparative genomic analyses to explore genome evolution and salidroside biosynthesis [27]. Subsequently, Zhang et al. (2025) assembled the first chromosome-level tetraploid genome of *R. kirilowii* (1.92 Gb), consisting of 40 homoeologous chromosomes and one sex chromosome, offering a valuable genomic resource for investigating polyploidization and comparative genomics in *Rhodiola* [28]. Most recently, Wang et al. (2026) and Miao et al. (2026) independently produced near T2T assemblies for *R. yunnanensis* and *R. macrocarpa*, respectively, constituting the highest-quality genome assemblies for the genus to date [29,30].

#### 2.2.1. Genome Assembly: From Draft Scaffolds to near T2T

Successive advances in sequencing technologies, assembly algorithms, and data integration strategies have brought about a dramatic enhancement in the quality of *Rhodiola* genome assemblies. This progression, from fragmented scaffold-level drafts to near T2T chromosome-scale assemblies, illustrates how long-read sequencing and Hi-C scaffolding can overcome the challenges posed by highly repetitive and heterozygous plant genomes.

Three key factors have collectively contributed to this progress. First, the transition from short-read (Illumina) to long-read sequencing platforms (PacBio and Oxford Nanopore) has been pivotal. Long reads can span highly repetitive regions that short reads cannot resolve, markedly improving contig continuity [31]. Second, the integration of Hi-C chromatin conformation data has facilitated chromosome-level scaffolding without requiring labor-intensive genetic maps, enabling contigs to be anchored, oriented, and ordered into pseudomolecules [32]. Third, assembly algorithms have advanced to address the specific challenges of *Rhodiola* genomes, including high heterozygosity, abundant repetitive elements, and in certain cases, polyploidy. Tools such as hifiasm are now capable of producing haplotype-resolved or subgenome-phased assemblies [33], whereas recent developments have further made possible T2T assembly using Nanopore Simplex reads, as exemplified by hifiasm [34] and HERRO [35].

These advancements are clearly evident in *Rhodiola* genomics: from the initial Illumina-based scaffold-level draft of *R. crenulata* [26], to the first chromosome-level assemblies for two diploid species using PacBio and Hi-C [27], to a tetraploid assembly with fully resolved homoeologous subgenomes [28], and finally to near T2T assemblies of *R. yunnanensis* and *R. macrocarpa* [29,30]. This progression highlights that the combination of long-read sequencing, Hi-C scaffolding, and advanced assembly algorithms is indispensable for resolving the genomes of non-model, highly repetitive, and recently radiated taxa such as *Rhodiola*.

#### 2.2.2. Expansion and Contraction: A Dual Evolutionary Pattern

Comparative genomic analyses have revealed a dynamic evolutionary pattern in *Rhodiola*: massive amplification of repetitive elements co-occurs with lineage-specific expansions and contractions of functional gene families, potentially reflecting adaptive trade-offs under high-altitude stress.

Across all sequenced *Rhodiola* genomes, transposable elements (TEs), i.e., mobile DNA sequences that can replicate or relocate within the genome, represent the predominant genomic fraction, ranging from 57.60% in *R. chrysanthemifolia* [27] to 71.94% in *R. macrocarpa* [30], with LTR retrotransposons, a major class of TEs that insert into the genome via an RNA intermediate, invariably accounting for the vast majority (46.25–67.58%) of the assemblies. Within the LTR class, *Gypsy* elements frequently outnumber *Copia*; these two major LTR families differ in their integration preferences and copy-number dynamics, particularly in larger genomes such as that of *R. kirilowii* [27], implying that *Gypsy* lineages may possess a greater propensity for proliferation or lower elimination rates, likely due to their preferential integration into heterochromatic regions [36]. LTR insertion estimates point to a sustained amplification burst starting at approximately 10 Mya and peaking between 1 and 2 Mya, a window coinciding with major phases of QTP uplift and Quaternary glacial–interglacial cycles [27]. This correlation is consistent with a possible contribution of environmental stress to TE de-repression, while the accumulation may also reflect that host silencing was temporarily overwhelmed or relaxed during rapid population expansions [37]. However, this temporal association does not establish causality.

Notably, the diploid–tetraploid comparison in *R. kirilowii* may indicate a synergistic interaction: although the tetraploid genome exhibits a somewhat lower proportion of TEs than its diploid counterpart, the absolute TE content increases considerably (by nearly threefold), which may reflect the combined effect of whole-genome duplication and subsequent TE amplification [27,28]. Polyploidization may intensify TE proliferation through genomic shock, i.e., the genomic disruption caused by whole-genome duplication, increased target-site availability, and relaxed purifying selection, i.e., the weaker removal of slightly deleterious insertions [38,39]. In turn, TE insertions may have remodeled regulatory landscapes by introducing cis-elements, promoting exon shuffling, and enabling ectopic recombination, thereby fostering genomic plasticity that may have promoted rapid alpine adaptation [40]. Thus, the repetitive architecture of *Rhodiola* genomes may not merely represent selfish DNA, but may instead constitute an active evolutionary reservoir that, together with polyploidy, may have contributed to genomic restructuring and lineage-specific adaptive traits.

Beyond repetitive element expansion, lineage-specific gene family dynamics may also have shaped the genomic landscape of *Rhodiola*. Here, “expanded” and “contracted” gene families refer to increases and decreases in gene copy number, respectively. Relative to the outgroup *Kalanchoe fedtschenkoi*, *Rhodiola* exhibits expanded gene families enriched in base excision repair and phenylpropanoid biosynthesis, and contracted families enriched in pollen recognition [27]. In *R. macrocarpa*, expanded families are enriched in DNA damage repair and telomere maintenance, while contracted families are associated with defense responses to bacterial and fungal pathogens as well as the ERAD (endoplasmic reticulum-associated degradation) pathway [30]. The contraction of defense-related families in *R. macrocarpa*, as inferred from comparisons with other *Rhodiola* species, is consistent with the possibility that reduced pathogen pressure at high altitudes may have relaxed selection on biotic defense pathways. These contrasting trajectories, namely the expansion of stress-response and repair systems alongside the contraction of biotic defense repertoires, may operate in parallel with TE-driven genomic amplification, representing a dual evolutionary pattern that may have contributed to the ecological success of *Rhodiola* in high-altitude environments. It is important to note that these comparative inferences are derived from studies with different species sampling, outgroup choices, and comparative designs. Therefore, the observed expansions and contractions should be interpreted as lineage-specific evolutionary patterns rather than as evidence for a single unified adaptive strategy.

### 2.3. Step Three: From Multi-Omics Integration to Functional Gene Discovery

High-quality nuclear genomes, together with transcriptomics, metabolomics, and microbiome sequencing, have propelled *Rhodiola* research into the multi-omics era. Genome assemblies provide a static genetic blueprint, whereas integrative multi-omics enables the dynamic interrogation of gene function, regulatory networks, and metabolite–environment interactions. This shift has been particularly transformative for two interrelated objectives: the elucidation of biosynthetic pathways for key specialized metabolites, with the salidroside pathway fully elucidated and the rosavin pathway partially elucidated, and the discovery of transcriptional regulators that orchestrate this metabolic network in concert with high-altitude stress adaptation. Here, “fully elucidated” denotes functionally validated enzymatic steps, whereas “partially elucidated” indicates that key steps remain supported by expression-based or heterologous evidence but have not been validated in planta.

#### 2.3.1. Biosynthesis of Salidroside and Rosavin: Validated Steps and Candidate Enzymes

The salidroside biosynthetic pathway was fully elucidated by integrating transcriptomics and metabolomics in *R. rosea*. Integration of multi-omics data revealed that a pyridoxal phosphate-dependent 4-HPAA synthase directly catalyzes the conversion of tyrosine to 4-hydroxyphenylacetaldehyde (4-HPAA) in a single enzymatic step [41], superseding the previously proposed two-step decarboxylation–deamination mechanism [42]. This earlier hypothesis was largely based on studies that had identified a tyrosine decarboxylase (TyrDC) from *R. rosea* [43] and a UDP-glucosyltransferase (UGT73B6) from *R. sachalinensis* [44], both implicated in the pathway. The complete pathway consists of three sequential enzymatic steps (Figure 3a): L-tyrosine is converted to 4-HPAA by 4-HPAA synthase, 4-HPAA is reduced to tyrosol by 4-HPAA reductase, and tyrosol is finally glucosylated to salidroside by a tyrosol—UDP-glucose glucosyltransferase. Functional validation through heterologous expression confirmed the successful reconstitution of the full pathway in host systems [41,45,46]. Thus, the core salidroside pathway in *R. rosea* is supported by functional evidence at each enzymatic step. The salidroside pathway has subsequently been further explored across diverse species. A comparative study of *R. crenulata*, *R. fastigiata*, and their natural intergrades demonstrated that salidroside content in *R. crenulata* (0.81%) is approximately 20-fold higher than in *R. fastigiata* [47]. Transcriptomic analysis revealed extensive differential expression in tyrosine metabolism and the phenylpropanoid pathways, and six candidate UDP-glycosyltransferases were identified, among which *RcUGT5914* showed species-specific expression, suggesting its potential role in elevated salidroside accumulation [47].

Beyond salidroside, rosavin and its related cinnamyl alcohol glycosides (collectively termed rosavins, which include rosarin and rosin) constitute another major class of signature bioactive compounds in *Rhodiola*, particularly in *R. rosea*, where they serve as key quality-control markers alongside salidroside [48]. Unlike salidroside, which is derived from the tyrosine-derived phenylethanoid pathway, rosavins originate from the phenylpropanoid pathway, with phenylalanine as the principal precursor [49]. The biosynthetic route for rosavins proceeds from phenylalanine to cinnamic acid (Figure 3a), then to cinnamoyl-CoA, cinnamaldehyde, and cinnamyl alcohol, which is subsequently glycosylated to produce rosin and rosavin [50]. The glycosylation step, mediated by UDP-glycosyltransferases (UGTs), plays a critical role in generating the structural diversity observed among rosavin derivatives [51,52]. Recent genome and transcriptome mining has identified candidate UGTs, particularly those belonging to the UGT91R subfamily, which are proposed to catalyze the addition of arabinose and glucose groups to the cinnamyl alcohol backbone, thereby yielding rosavin and its analogs [51,52]. Some of these UGTs have been functionally tested in an engineered *Escherichia coli* system [52], but their in planta functions in *Rhodiola* remain to be demonstrated. Therefore, the complete rosavin pathway remains only partially elucidated.

Thus, these two parallel pathways, originating from tyrosine and phenylalanine respectively, converge at the level of glycosylation while recruiting distinct UGT families. Their co-occurrence reflects a sophisticated metabolic network that directs carbon flux into two chemically discrete classes of bioactive compounds, potentially as an adaptive strategy to high-altitude environments.

Beyond pathway elucidation, the tissue-specific distribution of salidroside, rosavin, and other metabolites has been examined through combined transcriptomic and metabolomic approaches. In *R. rosea*, salidroside accumulates preferentially in the roots and rhizomes, which correlates with the organ-specific expression of biosynthetic genes such as *TyrDC* [43]. Similarly, rosavins also show tissue preference, with the highest levels typically found in rhizomes, although spatial patterns vary among species and chemotypes [53,54]. Transcriptome analysis across tissues in *R. imbricata* further revealed that NAC, bHLH, and ARF transcription factors (TFs) are the most abundant families, offering a valuable regulatory framework for understanding phenylethanoid and phenylpropanoid biosynthesis [49]. In a parallel comparative analysis, profiling of *R. crenulata* and *R. fastigiata* revealed that both tyrosol and total flavonoids attained their highest levels in *R. crenulata*, with distinct spatial distributions observed along different axial positions of the rhizome [47]. Collectively, these findings indicate the tissue-specific and genetically programmed coordination of phenylethanoid and phenylpropanoid biosynthesis in *Rhodiola*.

#### 2.3.2. Co-Expression Networks and Regulatory Factor Discovery

The identification of biosynthetic pathway genes represents only the first layer of understanding, whereas the regulatory mechanisms that govern their expression are equally critical. This has motivated recent genomic and transcriptomic studies to comprehensively survey the TF repertoires of *Rhodiola*, given the pivotal roles of TFs as master regulators.

The MYB family has attracted particular attention. A genome-wide survey in *R. crenulata* revealed 139 MYB genes based on conserved domain analysis [55]. Promoter analysis revealed *cis*-acting elements related to transcription, hormone signaling, and MYB binding. Importantly, a co-expression network was constructed based on the expression patterns of key genes in the salidroside biosynthetic pathway together with the identified MYB genes, generating predicted regulatory relationships that await functional validation. The bHLH family constitutes another major class of transcriptional regulators. Given that rosavin biosynthesis involves the phenylpropanoid pathway, which is regulated by MYB and bHLH TFs in other plants [56], it is reasonable to propose that these same TF families coordinate the parallel metabolic fluxes through the two branches, although this possibility awaits functional validation. Beyond MYB and bHLH, additional families including WRKY, NAC, and AP2/ERF have been implicated in the stress-responsive regulation of secondary metabolism and would also merit comprehensive functional characterization in *Rhodiola* [57].

The regulatory networks of specialized metabolism are closely intertwined with stress responses. High-altitude stresses, encompassing low temperature, UV radiation, hypoxia, and oxidative stress, concurrently challenge plant survival and influence metabolite accumulation (Figure 3b). The GPX gene family, which is essential for reactive oxygen species (ROS) scavenging, has been characterized in *R. crenulata* and may contribute to its stress tolerance [58]. Similarly, the RcUDPGT gene family, beyond its proposed role in salidroside biosynthesis, may also contribute to adaptive mechanisms [44,50]. Furthermore, both salidroside and rosavins exhibit antioxidant and stress-protective activities [59,60]. Their accumulation under stress conditions suggests that these pathways fulfill dual functions, producing bioactive metabolites while enhancing alpine stress tolerance (Figure 3b). This interconnection highlights the need to study stress responses and specialized metabolism as integrated systems.

#### 2.3.3. Plant-Microbiome Interactions

An emerging dimension of *Rhodiola* biology is the contribution of the plant microbiome to the modulation of specialized metabolism (Figure 3c). In *R. crenulata* grown at low altitude, inoculation with the fungal endophyte *Phialocephala fortinii* mitigated the decline in crucial metabolites [61]. Integration of transcriptomic and metabolomic data revealed that *P. fortinii* elicited differential regulation of 20 enzymatic genes associated with five distinct marker metabolite pathways, including those involved in carbohydrate and lipid metabolism, thereby indirectly supporting specialized metabolite production. Another endophytic fungus, *Trimmatostroma* sp. (ZPRs-R11), was reported to induce salidroside and tyrosol biosynthesis through signal cross-talk involving H_2_O_2_, NO, and salicylic acid [62]. Remarkably, some endophytic fungi isolated from *Rhodiola*, including *P. fortinii* Rac56, have been found to produce salidroside and *p*-tyrosol independently [63], suggesting the possibility of metabolic exchange between plant and microbes. Whether similar endophytic contributions exist for rosavin biosynthesis remains unexplored and constitutes a promising frontier. Broader microbiome studies conducted in *R. rosea* and *R. integrifolia* have begun to identify bacterial and fungal communities that are correlated with host metabolomic profiles [64]. The integration of microbiome data with plant transcriptomics and metabolomics opens new avenues for enhancing metabolite production through targeted microbiome engineering or microbial fermentation, offering a potential sustainable source of these valuable compounds.

## 3. Perspectives: From Elucidation to Design

Recent advances in genome assembly, adaptive evolution, and metabolic pathway elucidation in the genus *Rhodiola* have provided a solid foundation for deciphering the genetic architecture underlying its ecological adaptation and medicinal value. However, moving from “elucidation” to “design” necessitates the exploration of several frontier directions (Figure 4). Collectively, the following areas hold promise for advancing *Rhodiola* research from fundamental discovery toward engineering applications.

### 3.1. Pan-Genome Construction

Although multiple chromosome-level genome assemblies of *Rhodiola* species have been reported [26,27,28,29,30], most are confined to single species or limited numbers of individuals, which means that they cannot fully capture the full extent of the extensive interspecific and intraspecific genetic diversity within the genus. Furthermore, single reference genomes are inherently insufficient to represent the complete genetic repertoire of a species because of variable sequences and SVs that are not present in all individuals [65,66]. *Rhodiola* shows substantial variation in genome size, chromosome number, and SVs across species [27], features that may directly underpin differences in ecological adaptation, bioactive compound content, and ultimately “geo-herbalism” quality. Given the extensive hybridization and introgression documented within the genus [4,5], a single reference genome clearly cannot capture the entire gene pool. Consequently, future efforts should focus on the construction of a pan-genome that integrates multiple representative species and ecotypes or germplasms, thereby enabling the comprehensive identification of core and variable genes as well as regulatory SVs. Such a resource will enable a comprehensive analysis of the genetic basis of geo-herbalism and provide a solid genomic foundation for future quality improvement, molecular marker development, and targeted breeding.

### 3.2. Synthetic Biology Applications

The progressive elucidation of the salidroside pathway and the identification of candidate enzymes for rosavin biosynthesis have provided a solid enzymatic foundation for heterologous production, offering a sustainable alternative to harvesting wild plants. Over the past few years, substantial progress has been achieved in constructing microbial cell factories for the high-level production of these valuable compounds.

For salidroside, the complete pathway from tyrosine has been functionally reconstituted in both *Saccharomyces cerevisiae* and *Nicotiana benthamiana* [41,45,46]. However, early efforts encountered significant bottlenecks, particularly the competition for the glycosyl donor UDP-glucose (UDPG) between target glycoside synthesis and essential cell wall biosynthesis [67]. A recent systematic engineering strategy addressed this challenge by rewiring central metabolism through a thiamine diphosphate regeneration strategy, introducing the glycosyltransferase *RrU8GT33* from *R. rosea*, and applying cell wall engineering to redirect carbon flux toward salidroside production [67]. The final engineered strain achieved a record salidroside titer of 40.46 g/L in bioreactor fermentation, with a productivity of 0.24 g/(L·h) and a yield of 0.27 g/g glucose [67]. This work demonstrates the effectiveness of cofactor and cell wall engineering in optimizing glycoside production and provides a scalable strategy for the microbial manufacturing of high-value natural glycosides.

For rosavin, which features a more complex glycosylation pattern involving both glucose and arabinose residues, heterologous production has also seen considerable progress. By engineering *E. coli* to express a cinnamate:CoA ligase, a cinnamoyl-CoA reductase, and appropriate UDP-glycosyltransferases, the rosavin biosynthetic pathway was functionally reconstituted in an engineered *E. coli* system [52]. This provides proof-of-concept for heterologous rosavin production but does not establish the endogenous rosavin pathway in *Rhodiola*. Notably, four UGTs belonging to the UGT91R subfamily were found to catalyze the conversion of rosin into rosavin, with *SlUGT91R1* from *Solanum lycopersicum* exhibiting the highest activity. Through optimization of the UDP-arabinose supply pathway and employing fed-batch fermentation, the production of rosavin was boosted to 7539.1 ± 228.7 mg/L in a 5-L bioreactor, representing the first reported microbial production of this characteristic *R. rosea* component [52]. Thus, these findings point to the strong potential of the microbe-based production of rosavin for industrial application.

These achievements highlight the power of synthetic biology in mitigating the supply challenge of *Rhodiola*-derived bioactive compounds. Future efforts should focus on optimizing precursor supply, improving the catalytic efficiency of key enzymes such as UGT91R subfamily members, and developing cost-effective downstream processing strategies. The successful examples of salidroside and rosavin production also provide a valuable blueprint for the heterologous biosynthesis of other complex plant natural products.

### 3.3. Molecular Breeding and Precision Design

The genomic era has heralded new opportunities for the targeted breeding of *Rhodiola* and related medicinal species. To date, reports on marker-assisted selection and genome editing in this genus are still extremely limited [68,69]. Therefore, both CRISPR-based precision breeding and the targeted use of favorable SVs should be regarded as future possibilities rather than established applications in *Rhodiola*. Several gaps and limitations in current genomic data should also be acknowledged. These include the uneven quality of available genome assemblies, the absence of a *Rhodiola* pan-genome, limited functional validation of most candidate genes and regulatory relationships, the lack of efficient transformation and regeneration systems, the incomplete elucidation of the rosavin pathway, and the largely correlative nature of microbiome evidence. The available genomic and transcriptomic data allow for the comprehensive identification of molecular markers [47], including SNPs and InDels, that are closely associated with traits such as high bioactive compound content and enhanced stress tolerance, potentially expediting conventional breeding programs. Notably, CRISPR/Cas9 gene editing holds considerable potential for medicinal plant improvement. This technology has been successfully applied to modulate specialized metabolic pathways in diverse plants [70]. In *Rhodiola*, if efficient transformation and regeneration systems become available, CRISPR/Cas9 could potentially be used to knock out key enzyme genes in competing metabolic branches to redirect carbon flux toward target pathways, or to knock in strong promoter-driven key enzyme genes to increase metabolite accumulation [71]. Specific future directions could include pan-genome-guided marker-assisted selection, CRISPR/Cas9 knockout of competing branches such as phenylpropanoid side routes, and the overexpression of rate-limiting enzymes in the salidroside pathway. Moreover, the targeted use of beneficial alleles and favorable SVs identified through pan-genome analyses also remains a future prospect; once such variants are functionally validated and their effects are confirmed in relevant germplasm, they could potentially empower breeders to design more precise strategies for the targeted development of new varieties with high compound content and robust stress resilience. In addition, synthetic biology approaches for the heterologous production of salidroside and rosavin in microbial cell factories could help reduce pressure on wild populations and support sustainable supply. Realizing these future possibilities will require further technological development and functional validation, and could ultimately facilitate the transition of *Rhodiola* from wild harvesting to standardized and sustainable cultivation systems.

## 4. Conclusions

In summary, genomic research on *Rhodiola* has over the past decade progressively advanced from plastid to nuclear genomes and from structural characterization to functional discovery. Chloroplast genomes have provided a robust phylogenetic framework for resolving rapid radiation and reticulate evolution, while nuclear assemblies, now approaching T2T quality, have revealed dynamic patterns of expansion and contraction associated with transposable element bursts and gene family differentiation, providing hypotheses rather than mechanistic proof for high-altitude stress adaptation. Integrative multi-omics has further helped bridge the genotype–phenotype gap by fully elucidating the salidroside pathway, identifying candidate genes for rosavin biosynthesis, predicting regulatory transcription factor networks, and suggesting microbiome contributions to specialized metabolism. Collectively, these findings suggest that *Rhodiola* may achieve a coordinated evolution of stress tolerance and bioactive compound production, potentially through a combination of genomic conservation and localized innovation. These interpretations are primarily based on comparative, correlative, and multi-omics evidence, and functional validation of the underlying mechanisms remains necessary. Looking forward, pan-genome construction will be essential to capture the full spectrum of germplasm diversity, while synthetic biology and precision breeding offer potential pathways toward sustainable utilization. Ultimately, the continued synergy between multi-omics discovery and functional validation will be critical for deciphering the complex genetic architecture underlying both ecological adaptation and medicinal quality in this emblematic alpine genus.

## Figures and Tables

**Figure 1:**
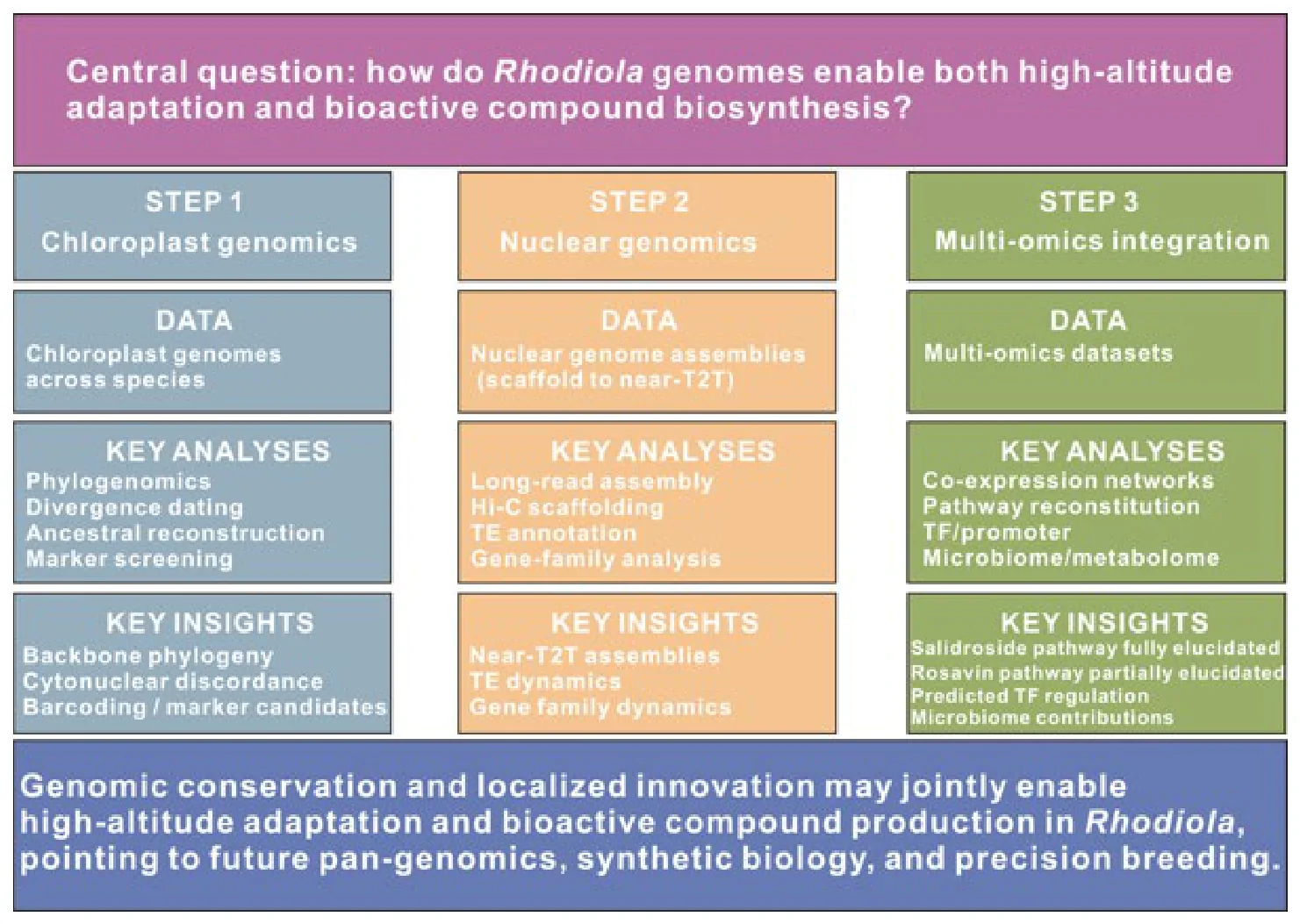
A three-step framework of *Rhodiola* genomics, from chloroplast and nuclear genomes to multi-omics integration.

**Figure 2:**
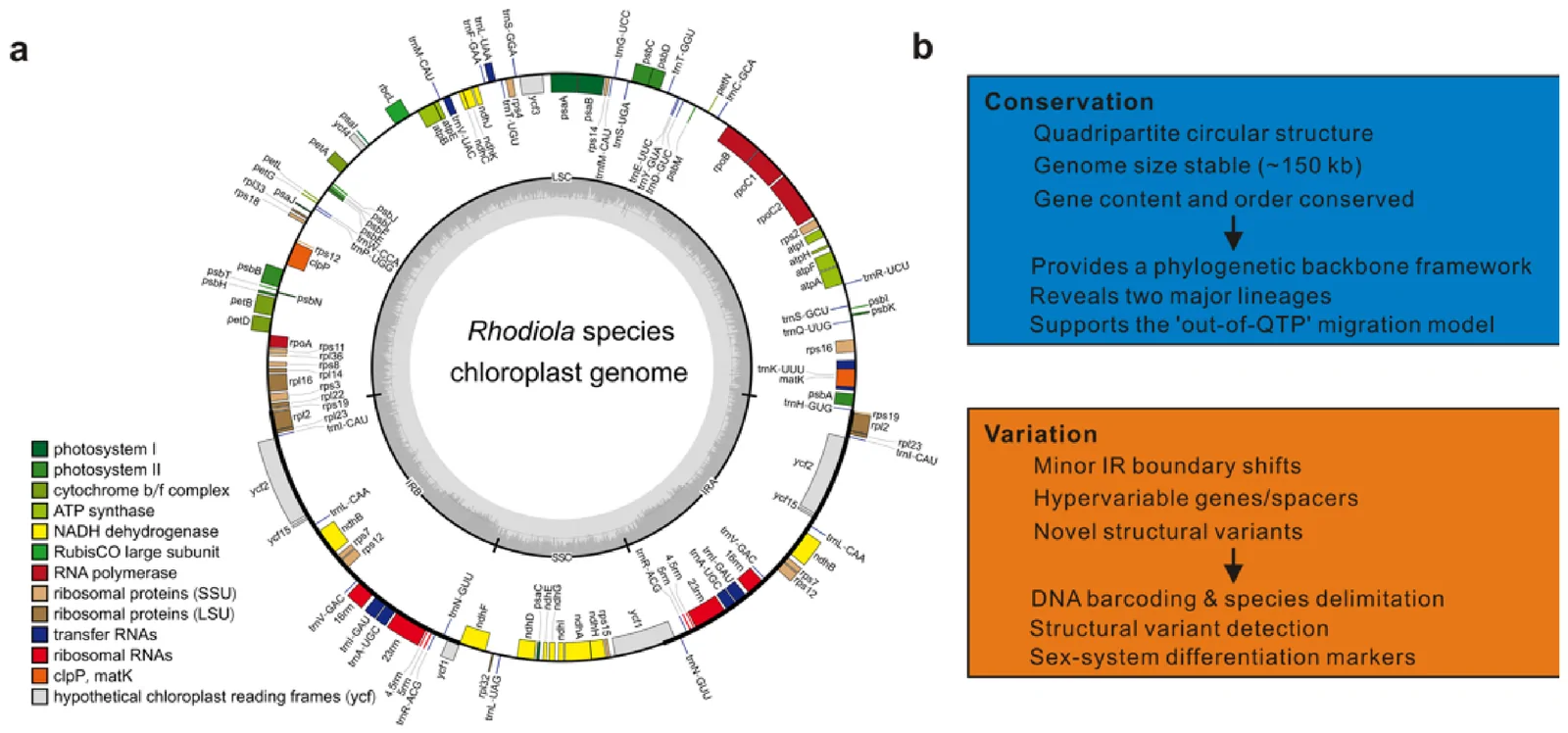
Chloroplast genome architecture in *Rhodiola*. (**a**) Representative circular genome map of a *Rhodiola* species. (**b**) Schematic summary of genomic conservation and variation with their respective applications.

**Figure 3:**
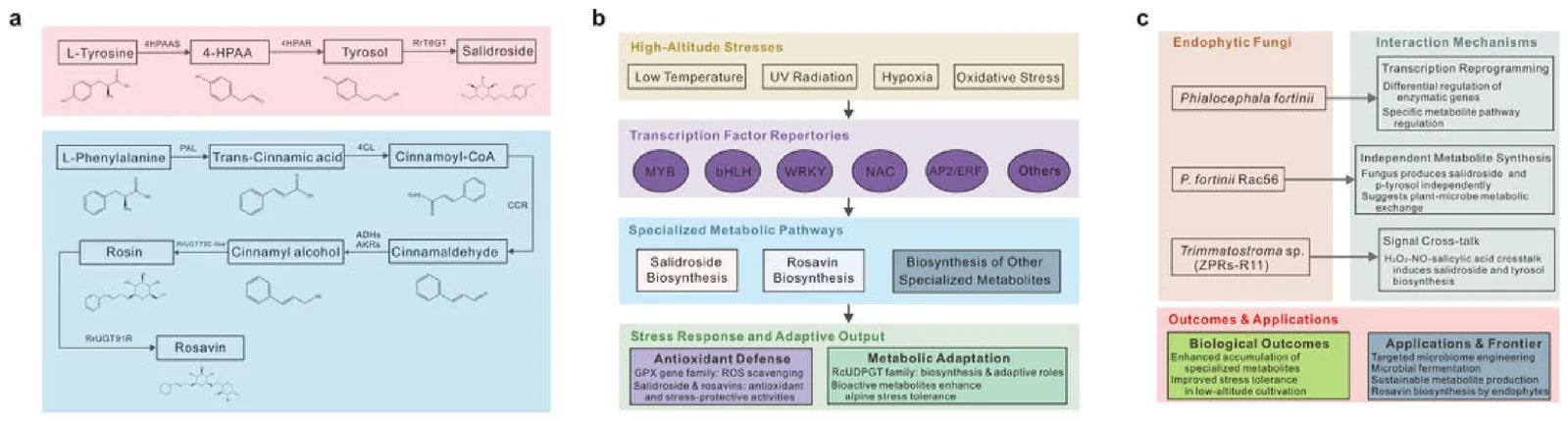
Multi-omics integration of specialized metabolism, transcriptional regulation, and plant-microbiome interactions in *Rhodiola*. (**a**) Biosynthetic pathways of salidroside (phenylethanoid) and rosavins (phenylpropanoid), showing key enzymatic steps and chemical structures. (**b**) Transcriptional regulatory networks connecting high-altitude stresses, transcription factor repertoires, and specialized metabolic pathways to antioxidant defense and metabolic adaptation. (**c**) Endophytic fungi modulate specialized metabolism via transcription reprogramming, independent metabolite synthesis, and signal cross-talk, with applications in microbiome engineering and sustainable metabolite production.

**Figure 4:**
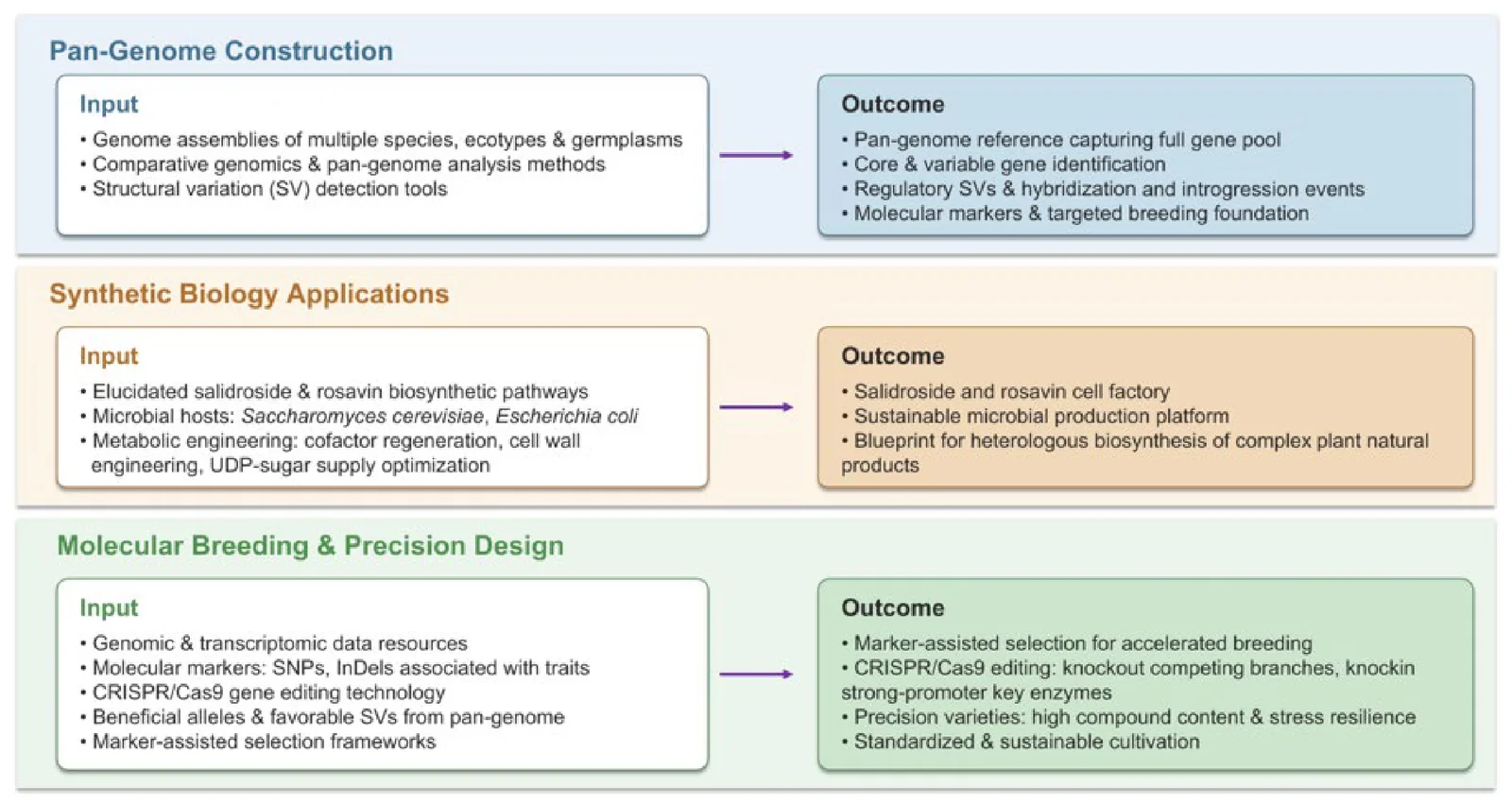
Perspectives: From elucidation to design in *Rhodiola*. Input-to-outcome frameworks for three translational directions: pan-genome construction, synthetic biology cell factories, and molecular breeding with precision design.

**Table 1:** Milestones of nuclear genome assemblies in *Rhodiola*.

Species	Ploidy	Chromosome No. (2n)	Assembly Level	Assembly Size (Mb)	Sequencing Technologies	Ref.
*R. crenulata*	Diploid	22	Scaffold	344.5	Illumina	[26]
*R. chrysanthemifolia*	Diploid	14	Chromosome	402.7	PacBio + Hi-C	[27]
*R. kirilowii*	Diploid	22	Chromosome	653.6	PacBio + Hi-C	[27]
*R. kirilowii*	Tetraploid	41 (40 autosomes + 1 sex chromosome)	Chromosome (haplotype-resolved)	1922.8	PacBio + Hi-C	[28]
*R. yunnanensis*	Diploid	22	Near T2T	643.3	ONT + Hi-C	[29]
*R. macrocarpa*	Diploid	20	Near T2T	727.5	ONT + Hi-C	[30]

## Data Availability

No new data were created or analyzed in this study. Data sharing is not applicable to this article.

## Abbreviations

The following abbreviations are used in this manuscript:

4CL	4-Coumarate:CoA ligase
4-HPAA	4-Hydroxyphenylacetaldehyde
ADH	Alcohol dehydrogenase
AKR	Aldo-keto reductase
AP2/ERF	APETALA2/ethylene-responsive factor
bHLH	Basic helix-loop-helix
CRISPR	Clustered regularly interspaced short palindromic repeats
ERAD	Endoplasmic reticulum-associated degradation
GPX	Glutathione peroxidase
Hi-C	High-throughput chromosome conformation capture
HIF-1α	Hypoxia-inducible factor 1-alpha
InDel	Insertion-deletion
IR	Inverted repeat
ITS	Internal transcribed spacer
LSC	Large single-copy
LTR	Long terminal repeat
MYB	Myeloblastosis
NAC	NAM/ATAF1/2/CUC2
NO	Nitric oxide
ONT	Oxford Nanopore Technologies
PAL	Phenylalanine ammonia-lyase
QTP	Qinghai-Tibet Plateau
ROS	Reactive oxygen species
SNP	Single nucleotide polymorphism
SSC	Small single-copy
SV	Structural variation
T2T	Telomere-to-telomere
TE	Transposable element
TF	Transcription factor
TyrDC	Tyrosine decarboxylase
UDP	Uridine diphosphate
UDPG	UDP-glucose
UGT	UDP-glycosyltransferase
